# Early Development of Locomotion in the Term Piglet Model: Does Size Matter?

**DOI:** 10.1093/icb/icad054

**Published:** 2023-06-12

**Authors:** Peter Aerts, Falk Mielke, Charlotte Vanden Hole, Merel J W Van Gorp, Chris Van Ginneken

**Affiliations:** Laboratory of Functional Morphology, Biology, University of Antwerp, Universiteitsplein 1, 2610 Wilrijk, Belgium; Movement and Sports Sciences, University of Ghent, Watersportlaan 2, Belgium; Laboratory of Functional Morphology, Biology, University of Antwerp, Universiteitsplein 1, 2610 Wilrijk, Belgium; Laboratory of Comparative Perinatal development, Veterinary Sciences, University of Antwerp, Universiteitsplein 1, 2610 Wilrijk, Belgium; Laboratory of Comparative Perinatal development, Veterinary Sciences, University of Antwerp, Universiteitsplein 1, 2610 Wilrijk, Belgium; Laboratory of Functional Morphology, Biology, University of Antwerp, Universiteitsplein 1, 2610 Wilrijk, Belgium; Laboratory of Comparative Perinatal development, Veterinary Sciences, University of Antwerp, Universiteitsplein 1, 2610 Wilrijk, Belgium

## Abstract

Intrauterine undernutrition in humans typically results in low birth weight ([small for gestational age] SGA) and delayed postnatal neuromotor maturation. Since SGA and intrauterine growth retardation are also common in domestic pigs, piglets are premised as models to study delayed motor development. Applied to the locomotor paradigm, however, questions emerge: (i) how to map the developmental time scale of the precocial model onto the altricial target species and (ii) how to distinguish size from maturation effects? Gait data were collected at self-selected voluntary walking speed during early development (0–96 hours postpartum; *pp*) for SGA- and normal ([appropriate for gestational age] AGA) piglets. Dimensionless spatiotemporal gait characteristics (according to dynamic similarity) become invariant already after 4 hours *pp*, suggesting rapid postnatal neuromotor maturation. Moreover, dimensionless gait data are largely identical for SGA- and AGA-siblings, indicating that primarily size effects explain absolute locomotor differences. This is further supported by (i) normalized force-generating capacity of limb muscles, (ii) joint kinematics (<10 hours *pp*), and (iii) normalized ground reaction forces (<5 days *pp*) being indifferent between SGA- and AGA- piglets. Furthermore, predictive modeling based on limb joint kinematics is unable to discern the majority of SGA- from AGA-piglets (<10 hours *pp*). All this leads to the conclusion that, although smaller than the AGA piglets in absolute terms, SGA-piglets mature (neuromechanically speaking) just like, and equally fast as their AGA littermates. Yet, it remains a fact that early SGA piglets are reported to be less mobile, less vital, and less competitive than their AGA siblings (even often die before day 3 *pp*). This conspicuous difference likely results from the energy level (blood glucose and glycogen) and its mobilization being considerably different between the piglet categories during early development.

## Introduction

Human fetuses are classified as small for gestational age (SGA) when on, or below, the 10th percentile of the fetal Body mass (BM) growth curve (Battaglia and Lubchenco, 1967; Beune et al. 2018; see also Kiserud et al. 2018). This implies that, at term birth, infants look 2–4 weeks younger, and have 5–10% less BM compared to the average neonate. Often, SGA infants suffer from Intra Uterine Growth Restriction (or retardation; IUGR), which is linked to severely increasing risks of morbidity and mortality with decreasing BM-percentile (Beune et al. 2018; Esih et al. 2022; Giabicani et al. 2018). Considering that, worldwide, 10–15% of all pregnancies can be classified as IUGR (up to 30% in developing countries), this is far from trivial (de Onis et al. 1998; Lee et al. 2013, 2017; McCowan et al. 2018).

IUGR infants are born with, amongst others, immature brains (fewer neurons, hypomyelination; for a review see: Baud and Berkane, 2019). This can, during further development, be linked to neuro-motor problems and deficits (problems in moving, maintaining balance or posture, etc.; Esih et al. 2022). Despite the above-mentioned high prevalence of IUGR cases, surprisingly few studies provide causal insights into the mechanistic aspects of these neuro-motor developmental problems. This calls for more fundamental (clinical) research to develop strategies for preventive care and appropriate treatment.

Clearly, both from the practical and (especially) the ethical point of view, research on human neonates and early infants is far from evident, even more so when these infants are classified SGA/IUGR. Therefore, both a representative and relevant motor paradigm and the proper model species are indispensable.

Locomotion is commonly considered a convenient option for this purpose. Its development proceeds along well-defined milestones that are readily and easily accessible and quantifiable (Adolph and Robinson, 2015). At the same time, however, locomotion is, despite its cyclical nature, a most complex motor task: propulsion must be combined with proper balance- and postural control (Adolph and Robinson, 2015; Hallemans et al. 2004, 2006). As such, extero- and proprioceptive feedback, reflexive response, cognitive anticipation, and adjustments, as well as the basic rhythmic feedforward drive, are all integrated into one and the same paradigm. Also, from a broader biological perspective, the focus on locomotion is relevant as it constitutes an important behavioral component of many ecological functions.

With regards to the proper model species, piglets are often put forward as the relevant substitute, as remarkable physiological and internal anatomical similarities exist with human infants (e.g., Mudd and Dilger, 2017; Odle et al. 2014). Moreover, and important in the context of SGA/IUGR, breeding selection for larger litter sizes has resulted in increased percentages (>20%) of SGA individuals, born in mixed litters together with otherwise appropriate for gestational age (AGA) littermates. Below a BM of 1.1 kg at term birth, early mortality increases sharply (Feldpausch et al. 2019), and these SGA piglets also suffer from increased morbidity. Prolonged farrowing and associated hypoxia are important factors in this, but the effect of the increased competition for nutrients among littermates cannot be underestimated (Baxter et al. 2008; Muns et al. 2016; Pandolfi et al. 2017). This competition not only happens during suckling (access to the teats), but already starts *in utero* during gestation, thus leading to varying levels of IUGR in SGA piglets (Vallet and Frekling, 2007; Vellet et al. 2011).

As for human term SGA/IUGR-infants, SGA/IUGR-piglets appear younger at birth (compared to the average BM of AGA piglets, the above-mentioned 1.1 kg boundary accords to a seeming difference of ∼2 weeks; Feldpausch et a., 2019). They are not only smaller but also show lower vitality (the level depending on the IUGR severity; see further), which leads to reduced competitiveness relative to their AGA littermates (Baxter et al. 2008; Litten et al. 2003; Muns et al. 2016; Pandolfi et al. 2017). Furthermore, they suffer from the same brain developmental problems and subsequent neuro-motor deficits as human SGA-neonates and infants do and show, when severely IUGR, conspicuous deviating head morphologies (“dolphin”-shape; Douglas et al. 2016; Hales et al. 2013; Riddersholm et al. 2021). As such, the term SGA/IUGR-piglet seems to represent the perfect model required to fill the above-mentioned knowledge gap in the neuro-motor maturation problems of term human SGA/IUGR-infants. However, before concluding this, some important issues must be addressed.

First, there is the problem of mapping ontogenetic time scales (Garwicz et al. 2009). Reaching the stage of independent walking takes approximately one year in humans, and even then, several weeks/months will pass before a somehow regular pattern emerges. Wildebeest calves, on the other hand, stand, walk, and even gallop with the herd within minutes after birth. In other words, the postpartum (*pp*) maturational time window may differ enormously between species (i.e., altricial versus precocial, respectively). Piglets, too, are precocial, and it should be questioned first what the relevant time window for their (loco-)motor maturation is. Second, it should be established in which aspects and to what extent SGA-piglets differ in the progress of their (loco-)motor maturation from the AGA littermates. If differences exist, it should (third) be questioned what aspects of these differences are just a matter of size (small *versus* appropriate size), which aspects are caused by altered (delayed) neuro-motor maturation, and which are the result of still other factors.

In this paper, we specifically address these questions. For this purpose, we integrate results of our former studies on the locomotion of term AGA piglets and SGA piglets (spatiotemporal gait data: Vanden Hole et al. 2017, 2018a; muscle morphometrics and fibre typing: Vanden Hole et al. 2018b, 2019a; energetics: Vanden Hole et al. 2019b) with new data on joint kinematics and single-limb dynamics. The interpretation of this new synthesis is matched against the outcome of Bayesian predictive modeling (Mielke et al. 2023).

## Methods

### Conceptual framework

**Fig. 1:**
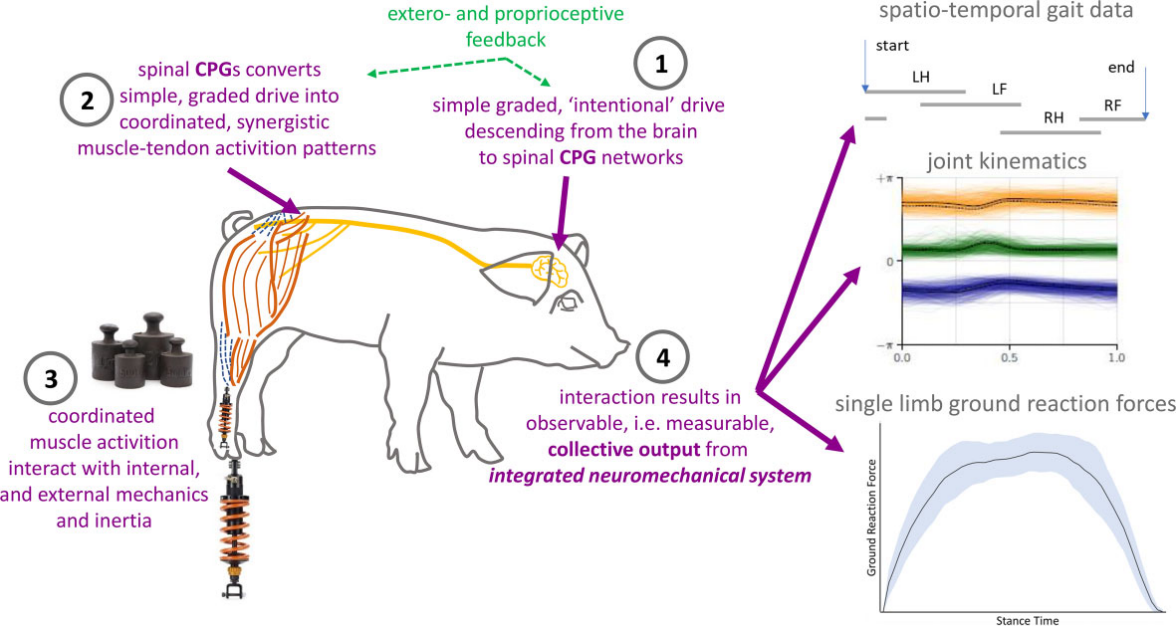
Conceptual framework. Control components of the locomotor system interact with internal and external mechanics (symbolized by the spring-dampers) and inertia (symbolized by the weights) thus providing the collective output from the integrated neuromechanical system (i.e., spatiotemporal gait data [represented by a theoretical footfall pattern], joint kinematics, and limb dynamics). See text for more explanations.

To answer the questions posed, we rely on the basic control scheme for voluntary locomotion (Fig. 1; e.g., Grillner, 2011; Grillner and El Manira, 2019; Nishikawa et al. 2007). Intentional locomotion initiates as a simple, graded drive, descending from the brain to the spinal Central Pattern Generators (CPGs; **1** in Fig. 1). These CPG networks convert this drive into coordinated, synergistic muscle-tendon activation patterns for all limb joints (**2** in Fig. 1). Both levels (brain and spinal) can be subject to external and peripheral feedback. It is, however, the interaction of the coordinated muscle activation with the internal and external mechanics and with the inertia of the system (**3** in Fig. 1) that finally results in the observable, in other words directly measurable, external collective output from the entire integrated neuromechanical locomotor system (**4** in Fig. 1): i.e., the spatiotemporal gait data, in the joint kinematics, and the single-limb ground reaction forces. As such, any observed ontogenetic change in any of the collective output variables of a specific locomotion task (for instance, walking at self-selected voluntary speed on level ground) must reflect alteration within the integrated neuromechanical locomotor system: (i) neuro-mechanical maturation at any level of the system (central or peripheral nervous system, muscle-tendon units, morphological aspects, etc.) and/or (ii) pure size effects.

When comparing the executions of specific (loco)motor tasks of similar but different-sized animals, size effects can be excluded by normalizing according to dynamic similarity principles (Alexander, 1977, 2003, 2005; Alexander and Jayes, 1983). In concept, motor tasks are dynamically similar when all linear dimensions (of the movements, as well as of the musculoskeletal system itself) can be scaled by a single factor $\alpha $ (i.e., in concept, animals should scale isometrically), all time-related aspects of the motor behavior can be scaled by another factor $\beta $, and all forces can be scaled by a third factor $\gamma $. This procedure generates the dimensionless expression of all relevant mechanical variables (morphometrics, kinematics, and dynamics). When the (loco)motor behavior for two different-sized animals is dynamically similar (i.e., identical behavior in dimensionless measures), tasks for the muscles (the “motors”) are similar too (i.e., work done per unit mass and per unit distance).

Reversed, however, the former reasoning also implies that, for identical motor tasks, any difference in collective output that remains after normalization according to dynamic similarity principles must reflect intrinsic neuromechanical changes. When framed in an ontogenetic context, such changes are likely to point to neuro-motor maturation. We opted for *self-selected voluntary speed* as the relevant identical motor tasks during the early development of SGA- and AGA-neonatal piglets (see also, for instance, Vaughan et al. 2003). The collective output of the integrated neuromechanical locomotor system (spatiotemporal gait data, joint kinematics, and single-limb dynamics) is assembled and normalized according to dynamic similarity principles to answer the specific research questions as formulated in the Introduction.

### Experimental approaches

Details on the specific in “Methods” section are presented in our former studies and will only be summarized unless the results presented here concerned additional and unpublished data. In this case, all required details will be provided. Institutional and national guidelines for the care and use of animals were followed, and all experimental procedures involving animals were approved by the Ethical Committee of Animal Experimentation, University of Antwerp, Belgium (approval numbers 2015–26 and 2017–25).

Spatiotemporal gait data (Vanden Hole et al. 2017; 2018a). Fourteen AGA piglets of normal vitality and 11 SGA piglets with low vitality from 7 litters (AGA = mass average ± 1 SD per litter; SGA < litter average–1 SD; normal vitality = normal respiration, standing/walking at birth; low vitality = labored respiration, no movement/standing) are selected for longitudinal follow up. Data are collected from lateral view video sequences (50 Hz) of voluntary walking (self-selected speed), recorded on the farm at 0, 1, 2, 6, 8, 24, 26, 28, and 96 hours *pp*. For the present study, stride (= cycle) length and frequency, step length (displacement during single-limb contact), duty factor (stance time/stride time), relative limb phasing (difference in footfall timings relative to stride time), and hip height at midstance are selected. Linear mixed models (classical statistics) were fitted to evaluate the effect of age (fixed factor) on the outcome variables. To account for the dependence between observations on littermates or within the same animal, random factors were included for sow and piglet (nested), plus random slopes for age and piglet (nested). The starting model was simplified using stepwise backward modeling. *Post hoc* analysis with Dunnett's correction was used for comparing normalized gait variables of the different age groups with the control age (i.e., 96 hours *pp*).

Joint angles (Mielke et al. 2023; additional analyses). For 50 AGA piglets ($\ge \ $0.8 kg at birth) and 8 SGA piglets ($< $0.8 kg at birth) within the age interval of 1 to 10 hours *pp*, stride cycles were selected from 10 min of continuous video recording (at the farm) of voluntary walking (self-selected speed) of each individual (cross-sectional study). Joint angle profiles are measured throughout the walking cycles. For the present study, shoulder, elbow, carpal joint, hip, knee, and tarsal joint angles are selected. Time profiles are transformed to the frequency domain (Fourier Series Decomposition, exponential form; eight harmonics retained for profile description; see also Mielke et al. 2020). This procedure provides for each joint the mean angle and the amplitude (general postural information) and 2 × 8 Fourier coefficients (profile “shape” information). For the six joints collectively, the coefficients yield information on the intralimb and interlimb coordination. A Principle Component Analysis is performed to reduce the variable count from 96 coefficients to 12 PCs. These components are considered coordination variables. Generalized linear modeling (Bayesian statistics) is used for the analyses.

Single-limb ground reaction forces (GRFs; unpublished data). Four Kistler® squirrel force plates were built in a walkway along, which AGA piglets and SGA piglets ($\ge $0.8 and $< $0.8 kg birthweight, respectively) walked at self-selected voluntary speed. Animals were recorded between day 1 and day 5 *pp* (cross-sectional setup). Only single-limb GRFs were used for further analysis. Although recorded at 1 kHz, force profiles were resampled to obtain a fixed number of 50 intervals per stance. Statistical parametric mapping (SPM; Pataky, 2012; www.spm1d.org), taking account of the dependence of samples at neighboring timepoints, was used to compare normalized force profiles (expressed in Body weights, BW; see further) between AGA piglets and SGA piglets (e.g., Pataky, 2012; Pataky et al. 2013, 2015; Vanrenterghem et al. 2012). To increase the number of useful samples, the first and last half of the stance phases were considered separately (note that spm1d-packageV4.0 enables to consider the two parts of the force profiles without neglecting the spatiotemporal dependence between neighboring time measurement points and the smoothness of the complete curve). Vertical and fore-aft ground reaction forces of fore- and hind-limbs were considered.

Force-generating capacity, fibre type composition, glucose, and glycogen levels (Vanden Hole et al. 2018b; 2019a; 2019b). Extensor muscles of the front- and hind-limb were dissected at one body side from AGA- and SGA-individuals at 0, 4, 8, and 96 hours *pp* (cross-sectional setup, at least 4 individuals per timepoint). Mass and mean fibre length were measured to determine the physiological cross-sectional area perpendicular to the muscle fibres, hence independent of contractile status on the fibre length (mass/density/fibre length; density = 1060 kg/m³ cf. Méndez and Keys, 1960). These areas were summed per limb and a muscle stress of 0.3MPa (cf. e.g., Williams et al. 2008) was used to estimate force-generating capacity. Samples for fibre typing were taken from the other body side. Blood glucose levels and glycogen levels in the liver were determined. Blood glucose was measured with a Onetouch Ultra glucose meter, immediately after the piglets were humanely killed using an overdose of anaesthetics and exsanguination. Glycogen concentrations were determined spectrophotometrically using the protocol by Theil et al. (2011) on a sample of liver tissue.

Bayesian Probabilistic Predictive Modeling (Mielke et al. 2023). Data collected for the analysis of the joint angles was used (see above). For each of the walking sequences, spatiotemporal data, neck angles, linear measures of the digitized segments, and sex were added to the database. Generalized linear models were trained on 34 variables (sex, 9 spatiotemporal, 12 postural, and 12 coordination variables) of 294 strides by AGA piglets to predict mass, size (relative measure based on Principle Component Analysis on segmental measures), and age of the individuals (reverse modeling) walking these specific strides. 35 different, randomly selected AGA strides were used for model validation. Finally, mass, size, and age were predicted for the individuals performing 39 strides of the 8 SGA-individuals (sampled in the 1 to 10 hours *pp* period) and compared with the actual mass, size, and age at the instant of sampling.

Body-part proportions (unpublished data). Cadavers of the specimens used for the GRF-measurements were stored in the freezer for further study when necessary. Heads (cut at the foramen magnum), front parts (in front of the diaphragm, minus the head), and hind parts (behind the diaphragm) of 7 AGA- and 10 SGA-specimens (aged between 1 and 5 days *pp*) were separated and weighed. Huxley’s allometric scaling model (*mass body part* = a *body mass*^b^) is fitted to the data (least squares fit), and the significance (biologically and statistically) of the exponent b is evaluated.

## Results and discussion

### Basic coordination patterns and maturation rate

The following results build on and reinterpret part of the longitudinal spatiotemporal gait data of Vanden Hole et al. (2017; 2018a) in the context of the framework of the present paper.

**Fig. 2:**
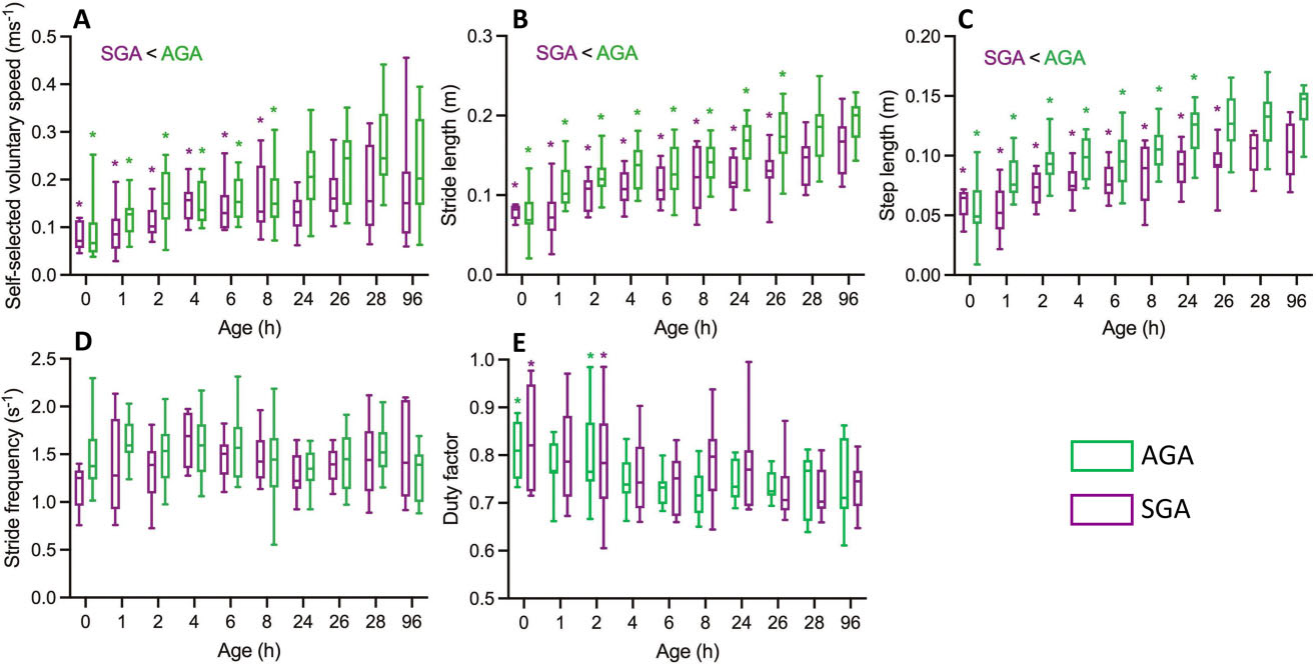
Spatiotemporal gait data (box and whiskers plots) for SGA- (purple) and AGA-(green) piglets when walking at self-selected voluntary speed during early development. When piglet categories differ, this is indicated above the graphs (i.e., in case of speed, stride length, and step length). Asterisks refer to significant differences with the variable considered as measured at the reference age of 96 hours pp.

To estimate the time window in which piglets are subject to locomotor development, we used the spatiotemporal gait characteristics as collected for the 14 AGA- and 11 SGA-piglets aged between 0 and 96 hours *pp* (cf. “Methods” section). In absolute terms (m/s), the self-selected voluntary speed increases slightly during early development (Fig. 2A). SGA piglets walk slightly more slowly than the AGA piglets, but from day 2 onward (24 hours *pp*), voluntary walking becomes indifferent from the reference at 4 days of development (96 hours *pp*; Fig. 2A). This speed increase is caused by the stride- and step lengths becoming larger with age. SGA-piglets use shorter strides and steps than AGA piglets, and differences with the reference age remain until 24 hours, respectively, 26 hours pp (Fig. 2B and C). Stride frequency and duty factor are similar for both categories, and only duty factor differs from the 96 hours *pp* reference at age 0 and 2 hours (Fig. 2D and E). Relative limb phasing does not change with age and is indifferent to the piglet categories. Absolute spatiotemporal stride characteristics suggest that, during early development (<96 hours *pp*), locomotor maturation happens fast after birth, essentially within one to two days.

**Fig. 3:**
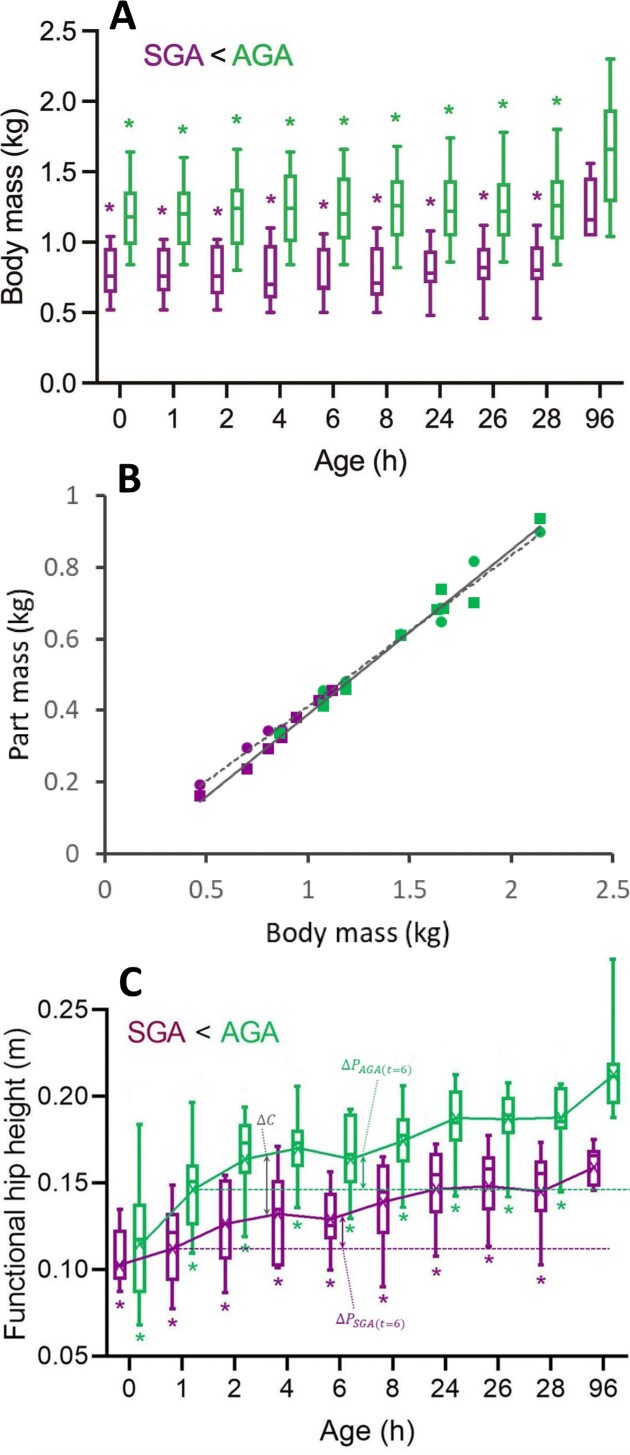
(A) BM as a function of pp age. Color codes and indication of significant differences are as in Fig. 2. (B) Masses of the front (circles; dashed curve; 0.41BM^1.02^) and hind (squares; solid curve; 0.38BM^1.16^) parts of the piglets as a function of BM (see text for more explanation). (C) Functional hip height as a function of pp age . $\Delta {\mathrm{C}}$ (double headed arrow) refers to the inter piglet category difference, $\Delta {\mathrm{P}}$ (double headed arrows) to the developmental time dependent postural change in AGA and SGA (see text for more explanations). Color codes in panels A–C and indication of significant differences in panels A and C are as in Fig. 2.

Prior to examining which of the ontogenetic changes can be attributed to size, it is useful to quantify how size actually differs between piglet categories and ages. Fig. 3A shows a clear, consistent difference (of ∼50%) in BM between the SGA- and AGA-piglets. Moreover, within each piglet category, BM hardly changes over the first 28 hours *pp* (Fig. 3A, Vanden Hole et al. 2018a). Also, when considering a BM-range present in the time window, including the reference age of 96 hours *pp*, front and hind part masses (see “Methods” section) constitute nearly fixed fractions of BM (Fig. 3B; masses respectively equal to 0.41BM^1.02^ and 0.38BM^1.16^; in the case of the front parts, the exponent is effectively indifferent from 1), and growth patterns of SGA- and AGA-piglets appear very similar (Fig. 3C). Based on all this, we presume that the data can be sufficiently well approximated by an isometric growth model, which is an assumption and requirement of normalization according to the dynamic similarity principle (see conceptual framework). Note that, even when applied to animals differing considerably more in body build and posture than the present piglets (e.g., different taxa, different ontogenetic stages), normalization according to dynamic similarity yields very useful and reliable results (e.g., Aerts et al. 2000; Alexander, 1977; Alexander and Jayes, 1983; Bullimore and Donelan, 2008; Druelle et al. 2017; Vaughan and O'Malley, 2005).

For the purpose of scaling motor behavior according dynamic similarity, selection of a mechanistically relevant dimensional scaling variable is necessary. We opted for a *dynamic* measure for functional limb length *during* walking, i.e., hip height *h* at midstance (with, in practice, the distance “hoof—tailbase” as a proxy). This choice is based on the rationale that this measure directly links with the inverted pendulum mechanism of the walking gait (cf. Alexander, 1977, 2003, 2005; Alexander and Jayes, 1983). Scaling the temporal aspects is further obtained by dividing by $\sqrt {h/{\boldsymbol{g}}} $ (with ${\boldsymbol{g}}$ gravitational acceleration; e.g., Hof, 1996), while forces are expressed in BW.

As mentioned earlier, conceptually, any difference in the collective output of the musculo-skeletal system that remains after normalization according to dynamic similarity principles should reflect intrinsic neuro-motor changes. However, since the developmental pattern of hip height appears a bit more intricate than that of BM (see Fig. 3C and compare with 3A), a cautious interpretation of the observed differences and changes in *h* is needed.

Firstly, as for BM, a rather consistent difference between the two piglet categories exists in the considered early developmental time window (<28 hours *pp*). Except at birth (*i.e.*, 0 hour *pp*) when hip heights are similar for both piglet categories, dynamic hip heights at midstance are consistently ∼30% larger in AGA piglets ($\Delta C\ $in Fig. 3C). According to the direct skeletal measurements of limb length presented in Vanden Hole et al. (2018b) and conform to the hardly changing BM, the major part of this $\Delta C$ (about 80%) can directly be attribute to the larger size of the AGA piglets. The remaining difference in hip height at midstance (about 20% of $\Delta C$) thus results from a consistently somewhat more flexed limb posture in SGA-piglets (see also below).

Secondly, and contrary to BM, there is also a developmental change of the hip height *h* at midstance in the considered time window (1 hour–28 hours *pp*; $\Delta P$_SGA(t)_ and $\Delta P$_AGA(t)_ in Fig. 3C). This change is remarkably similar for SGA- and AGA-piglets, both in the relative magnitude and in the shape of its pattern (Fig. 3C). Because of the nearly constant BM in this time window, the observed increase in *h* reflects primarily increasing limb extension, not morphological limb growth (as also evidenced by the direct skeletal limb length measurements at 2, 4 and 8 hours *pp* by Vanden Hole et al. 2018b). For both piglet categories, the hip height *h* at midstance increases by about 28%, most of which by 8 hours *pp*. After day 1 *pp*, no further change in limb posture is observed in the considered time window.

Whereas the first-mentioned consistent difference in hip height between SGA- and AGA-piglets (i.e., $\Delta C$) does reflect a pure size difference (including the small, but systematically more flexed limb posture of SGA), the postural change ($\Delta P$_(t)_) reported above, similar for SGA- and AGA-piglets, does not. As a result, hip height *h* at midstance, intended to be used as a (mechanistically relevant) normalization variable to remove size effects according to dynamic similarity, is to some extent biased by maturational effects reflected in a gradually extending limb. Therefore (1), *within piglet categories*, differences (with the reference age of 96 hours *pp*) that have disappeared after normalization cannot be claimed to be exclusively the result of the removal of size-effects and differences that remain likely reflect maturation effects other than changing limb posture. This holds primarily true for the very first hours of development (< 24 hours *pp*), when most of these postural changes occur (cf. above). What fraction of this is size removal and what fraction is due to masking the maturational postural change cannot be deduced from the present analysis. [Notice that the more flexed limb posture in the early stages is probably generic in mammals (see, for instance, Dewolf et al. 2021; Peters, 1983; Shapiro and Young, 2012; Sutherland et al. 1980; Thelen et al. 1984). This phenomenon (sometimes referred to as “flexor dominance”) is attributed to a temporary, early deficit in extensor strength].

However (2), given both the consistency of size differences between SGA- and AGA-piglets and the large resemblance of the pattern of postural changes (cf. above), differences *between piglet categories* that have disappeared after normalization were truly only there because of the size difference the between SGA- and AGA-piglets.

**Fig. 4:**
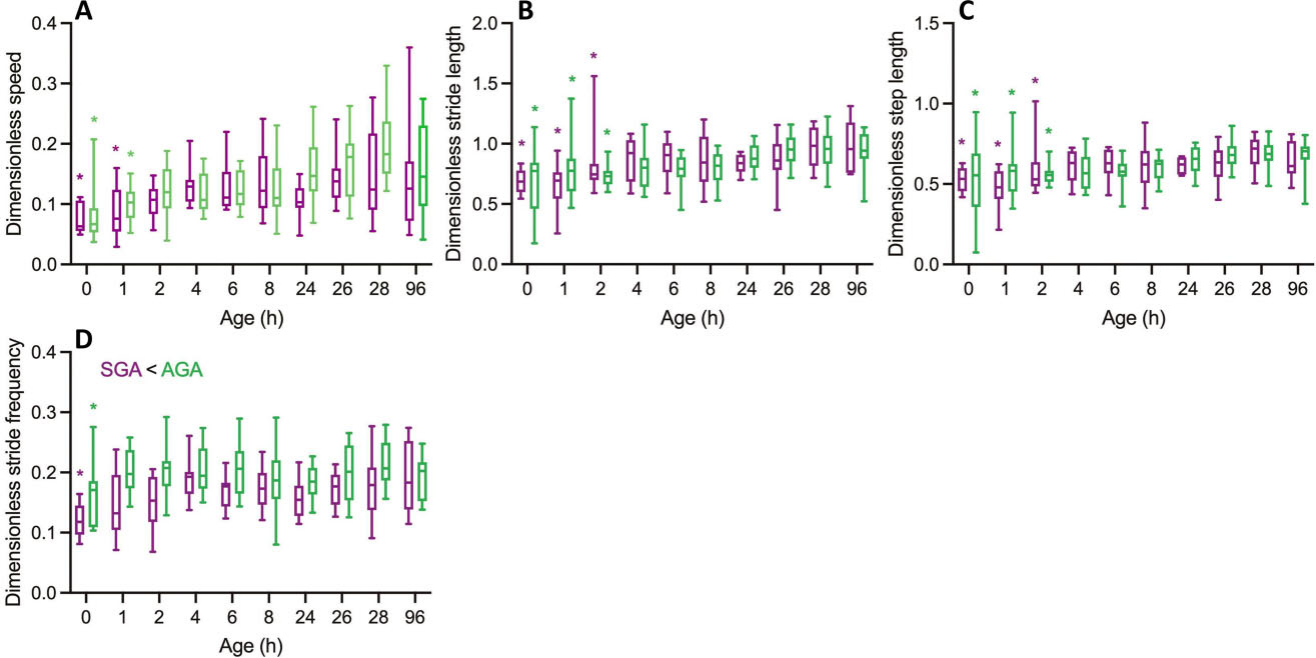
Dimensionless spatiotemporal gait data (normalized according to dynamic similarity; see text) for SGA- and AGA-piglets when walking at self-selected voluntary speed during early development. Color codes and indications of significant differences are as in Fig. 2.

What follows are the results of the normalization. Only below 2 hours *pp*, dimensionless voluntary walking speed ($V/\sqrt {h{\boldsymbol{g}}} $; with *V* the absolute walking speed) differ from that of the reference age of 96 hours *pp* (Fig. 4A). Moreover, dimensionless self-selected walking speeds do not differ between SGA- and AGA-piglets. Similarly, dimensionless stride- and step lengths ($L/h$; with *L* the absolute stride- or step length resp.) are indifferent to SVA- and AGA-piglets, while statistical differences with the reference age are only apparent before 4 hours *pp* (Fig. 4B, C). Except immediately *pp*, dimensionless stride frequency ($F/\sqrt {{\boldsymbol{g}}/h} $; with *F* the absolute frequency) is indifferent from that at 96 hours *pp* for both SGA- and AGA-piglets, yet on average, SGA-piglets seem to walk at slightly lower dimensionless frequencies. Given the identical dimensionless velocity and stride length for both categories, the latter difference is probably a coincidence of the large but biologically logical variability during early development. Duty factors and relative phases, dimensionless as such, have already been dealt with (see above).

Relying on the cautious, extensive discussion of the normalization variable provided above, these dimensionless results still leave us to conclude that the *early neuro-motor development of AGA- and SGA-piglets proceeds largely identically*. Moreover, the *neuro-(loco)motor maturation apparently proceeds rather rapidly*, i.e., in <4 hours *pp*

### Joint movement profiles and inter- and intralimb coordination

The following results rely on the additional statistical analysis (see “Methods” section) of the joint angle profiles throughout locomotor cycles used and discussed in Mielke et al. (2023) for predictive modeling purposes. As argued above, stride (i.e., cycle frequencies) hardly change during early development or between piglet categories. Therefore, for the present comparison (with focus on the time window from 1 to 10 hours *pp*), the progress of time throughout a cycle can safely be expressed as (dimensionless) fractions of cycle duration. Joint angles (expressed in radians) are dimensionless as such.

**Fig. 5:**
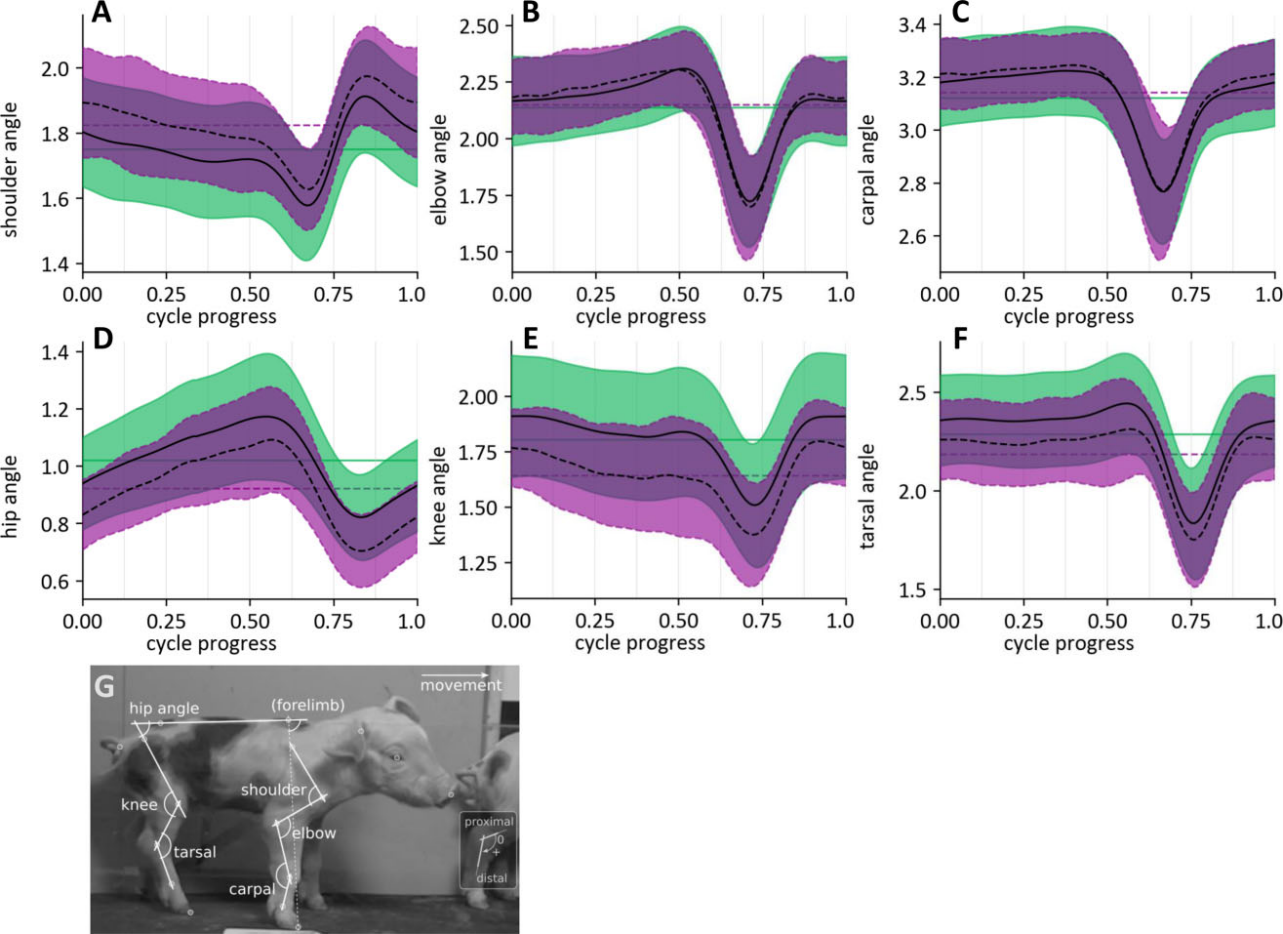
limb joint angle kinematics (in radians; ${\mathrm{\pi }}$= completely extended joint, 0 = completely flexed joint) of SGA- and AGA-piglets (measured as show in panel G; adapted from Mielke et al. 2023) when walking at self-selected voluntary speed. Relative 0 and 1 (*x*-axis) refer to consecutive touchdowns of the limb considered (frontlimb = panels A–C; hindlimb = panels D–F). Color codes are as in Fig. 2. Dashed straight horizontal lines indicate the temporal average of the joint angle profiles (AGA: green, SGA: purple). The dashed curve is the average SGA-joint profile (+ range in purple), the solid curve the average AGA-joint profile (= range in green).

**Fig. 6:**
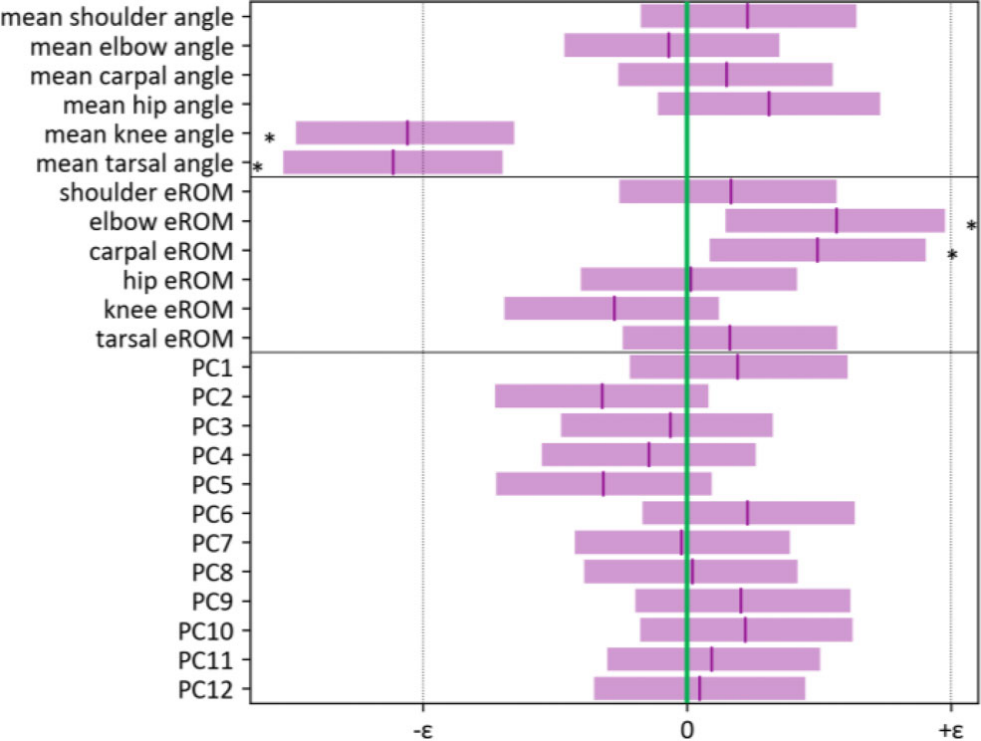
Statistical comparison of positional-(mean angles and RoMs) and coordination (PC1-12) variables between AGA piglets (green reference line) and SGA piglets (mean ± SD) as revealed by Bayesian linear statistical modeling. Asterisks indicate significant differences; ${\mathrm{\varepsilon }}$ = residual variance.

In addition to spatiotemporal collective variables, differences and maturation of intralimb coordination could appear at the joint level. Figure 5 shows the joint angle profiles for the front- and hind-limb for AGA- and SGA-piglets within the 1 to 10 hours *pp* developmental time window. Zero-time and time “1” accord to consecutive touchdowns of the limb. Qualitatively, profile shapes are very similar for both piglet categories. The average angle of the joint oscillations differs to some extent for shoulder, hip, knee, and tarsal joint angles, reflecting the abovementioned configurational differences of the functional limb length during early development (see above). However, only the average knee and tarsal joint angles of the cycles of the SGA piglets differ significantly from those of the AGA piglets (about 11° and 6° respectively; Fig. 6). This links up with the findings concerning the differences in dynamic hip height and hind limb posture as discussed above. Similarly, only for the elbow and carpal joint, ranges of motion seem to differ (i.e., somewhat larger in SGA) between piglet categories (Fig. 6). Interlimb and intralimb coordination SGA piglets are indifferent from that of AGA piglets (cf. coordination variables, Fig. 6). Based on these results (free of size effects), it seems that also joint kinematics and coordination of SGA- and AGA-piglets are largely identical during early development.

### Limb dynamics

As mentioned in “Methods” section, the results presented in this section were not published before. Vanden Hole et al. (2017, 2018a) showed that normalized stance duration is identical for SGA- and AGA-piglets and is only different from the stance duration at the reference age of 96 hours *pp* immediately after birth (time “0”). Therefore, stance time can be expressed for all cycles as a fraction (with zero-time equal to touch down and time “1” equal to lift-off of the limb considered).

**Fig. 7:**
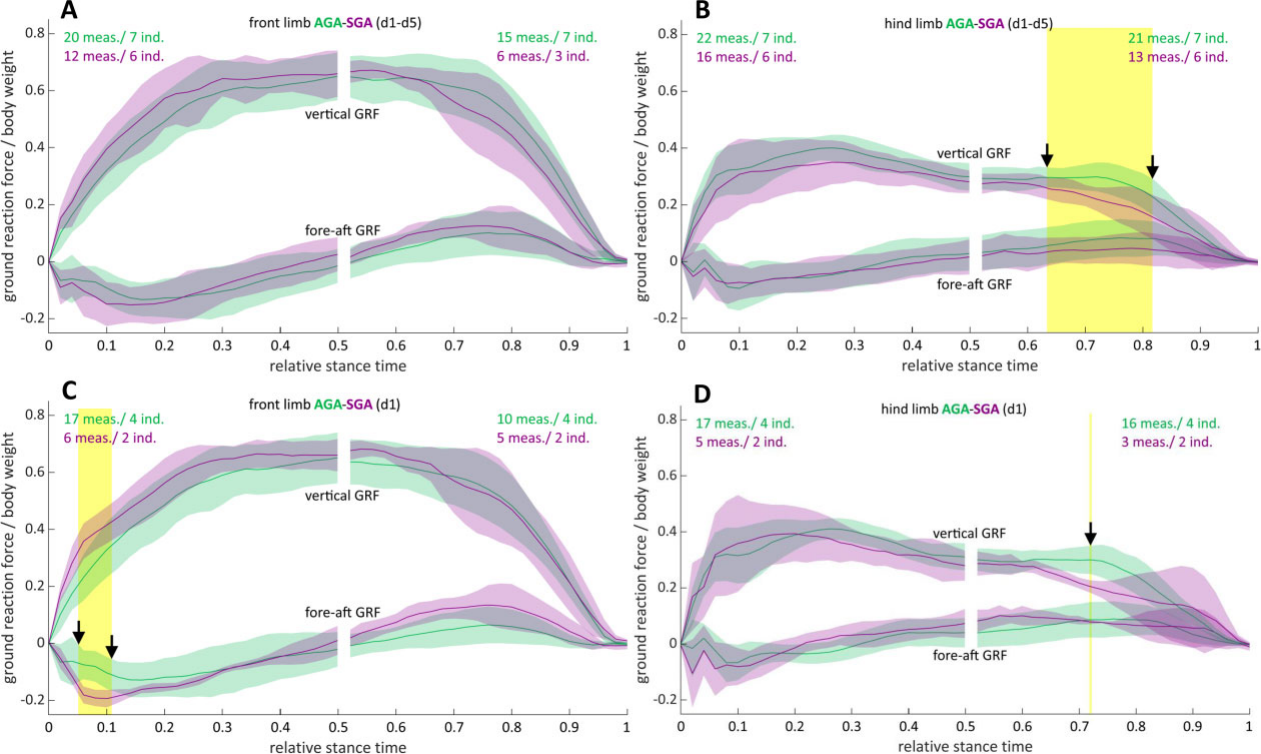
Single-limb vertical, and fore-aft ground reaction forces (averages + ranges; expressed in BWs as a function of relative stance time) of the frontlimb (A and C) and the hindlimb (B and D) of SGA- and AGA-piglets walking at self-selected voluntary speeds. Color codes are as in Fig. 2. The number of cycles (# meas.) as obtained from the number of individuals (# ind.) are indicated. Yellow shaded areas represent significant differences between the SGA- and AGA-profiles for the profiles (vertical or fore-aft) marked by the arrow heads.

Figures 7A and B present the normalized sagittal single-limb GRFs (medio-lateral forces not considered) for the front and hindlimb cycles (covering the entire 96 hours period) of SGA- and AGA-piglets. Only late in stance (from ∼65 to 80% of stance), small differences in the vertical components of GRF profiles of the hindlimb could be detected (yellow shaded region), with SGA-piglets generating somewhat lower GRFs. Focussing on the recordings collected during day 1 *pp* only (Figs. 7C, D; remember that maturation most likely proceeds before 4 hours *pp*, cf. above), these differences in the vertical components are no longer apparent (except for a biologically meaningless small fraction of the stance time; cf. Fig. 7D), but this is probably an effect of the limited number of available recordings. Yet fore-aft forces of the frontlimb show in this early developmental period now some small differences between the piglet categories during a brief period at the beginning of stance (yellow shaded region).

In general, most of the dynamic loading during walking is taken by the forelimbs during early development, and most differences between SGA- and AGA-piglets (if present) happen at the level of the hind limbs, generating somewhat less force (or being unable to provide the force as AGA-piglets) late in stance. Yet, these differences remain small. Hence, in general, it can be concluded that (size effects excluded), during early development, single-limb dynamics are largely identical in both piglet categories and that these dynamics don't change with age.

### Force-generating capacity and fibre composition of the limb extensors

The following cross-sectional data are summarized from Vanden Hole et al. 2018b, 2019a).

**Fig. 8:**
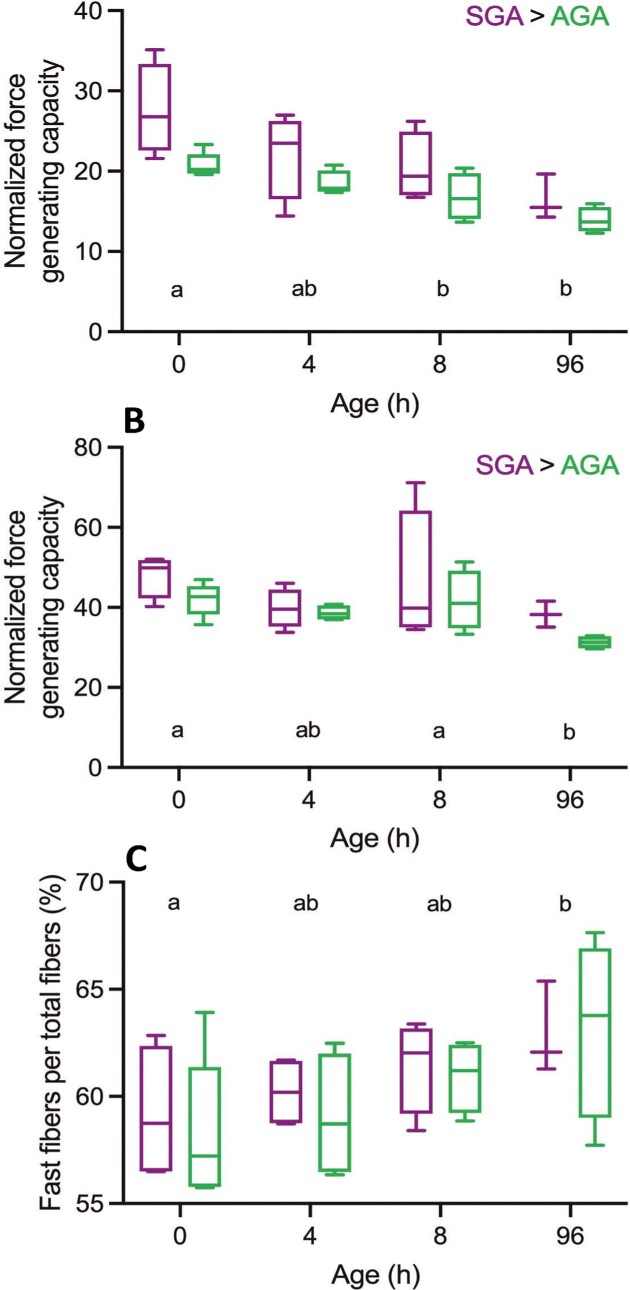
Force-generating capacity (in BW-s) for front (A) and hind (B) limbs of SGA- and AGA-piglets as a function of pp age. (C) represents the fibre composition (% fast fibres) for the front limb (hind limb %-s are identical for ages and piglet categories). Significant differences between piglet categories are shown above the graphs. Indifferences are indicated with the letter-labelling (same letter = indifferent).

The component of the integrated neuromechanical locomotor system that finally powers the collective output, as presented and discussed above (spatiotemporal gait data, joint kinematics, and limb dynamics), are the muscles. Overall, SGA-piglets have a somewhat higher normalized force-generating capacity (physiological cross-sectional area * 0.3 MPa/BW) than AGA-piglets in both the frontlimb and hindlimbs (see Figs. 8A and B). Moreover, individuals seem to be relatively stronger at birth than at the reference age of 96 hours *pp*. The fibre type composition (fast-slow) is identical for SGA- and AGA-piglets and does not change during early development (see Fig. 8C). Apparently, at the muscular level (and size effects excluded), SGA piglets do equally well (or even slightly better) than their AGA littermates.

### Size and BM: Bayesian probabilistic predictive modeling

**Fig. 9:**
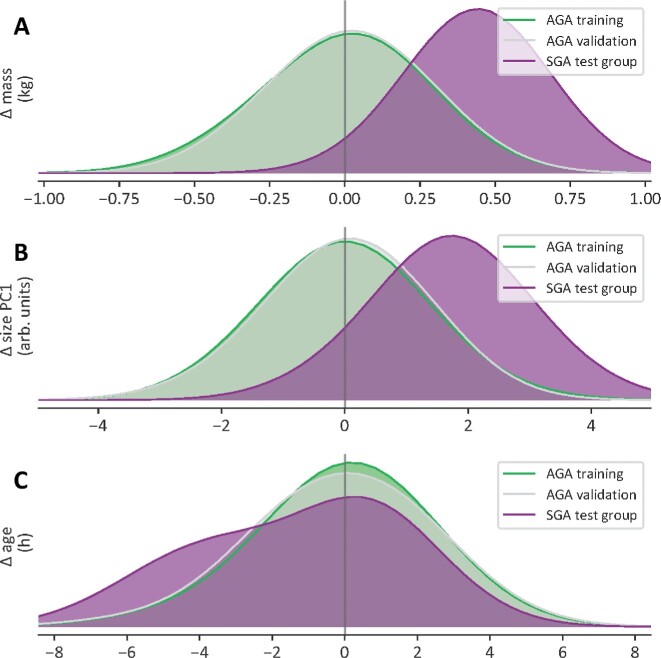
Differences between the real mass (A; kg), linear size (B; arbitrary measures), and age (C; hours pp) between values as predicted by Bayesian predictive modeling and the actual masses, sizes and ages of the SGA- and AGA-piglets. Color codes as in insets. See text for more explanation.

Taking all the above into consideration, it appears that, although SGA-piglets are, in absolute terms, definitely smaller than the AGA-piglets, they mature neuromechanically speaking just like and equally fast as their AGA-littermates. This conclusion seems to be confirmed by an alternative approach (Bayesian probabilistic modeling) in which mass or size are predicted from (normalized) locomotor kinematics (*i.e*., from collective output of the entire integrated neuromechanical locomotor system; *cf*. above). As mentioned in the “Methods” section, these models are trained and validated on data (kinematics and coordination) from stride cycles executed by AGA piglets in the 0 to 10 hours *pp* period (cf. Mielke et al. 2023). Next, the mass or size of *imaginary* piglets are predicted using kinematics and coordination extracted from SGA-stride cycles (executed 0–10 hours *pp*). These predictions are then compared with the masses and sizes of the *real* SGA-piglets that executed the strides that served as model input. Systematically, the model predicts the imaginary piglets as heavier and larger than the real SGA piglets (i.e., imaginary piglets are “AGA-like”; see Figs. 9A, B). In other words, the computational classifier does not find anything special about the SGA-data, and classifies them “as normal,” which can be interpreted as evidence for neuromechanical similarity to matching AGA.

### Stagnation in neuro-motor performance due to energy-deficits

Although the above findings seem to suggest that SGA-piglets are just only smaller than their AGA-littermates, it remains that they are found to be less mobile, less vital, less competitive, more vulnerable, and even often die before day 3 *pp* (Feldpausch et al. 2019; Quiniou et al. 2002). This morbidity (and mortality) is probably reflected in the outcome of another Bayesian predictive model, similarly trained on AGA-kinematics and coordination, predicting age *pp* (instead of size or mass) of *imaginary* SGA-piglets from stride cycle data of *real* SGA-individuals (executed 0–10 hours *pp*). For cycles from SGA-piglets older than the 4 hours *pp* boundary (i.e., the age above which no further “size-free” maturation differences could be detected between SGA- and AGA-piglets), the age is underestimated (with 2 to 5 hours *pp*) in 75% of all cases (see Fig. 9C; cf. Mielke et al. 2023). In other words, most of the strides of the older (real) SGA-individuals (5 to 10 hours *pp*) seem to have retained the neuro-motor level otherwise characteristic for the younger AGA-piglets.

**Fig. 10:**
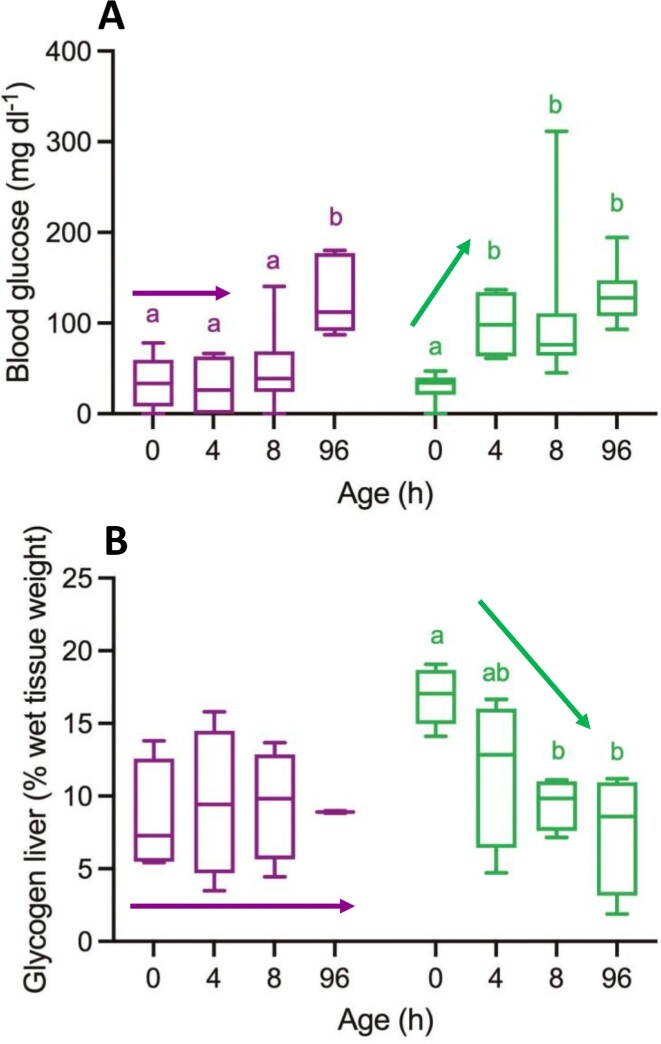
Blood glucose levels (A) and liver glycogen (B) in SGA- and AGA-piglets during early development. Color codes are as in Fig. 2. Identical letter-labels show indifferences within the piglet categories. Arrows indicate qualitatively the major differences in the developmental changes of the concentrations (no change = horizontal) between SGA- and AGA-piglets. See text for more explanations.

This stagnation in neuro-motor performance can probably be explained in terms of energy deficits. Fig. 10 is based on the results presented by Vanden Hole et al. (2019b). At birth, blood glucose levels (in mg/dl) are identical for SGA- and AGA-piglets (Fig. 10A). This picture, however, changes very rapidly. At 4 hours *pp* (the maturation boundary cf. above), this level has more than doubled in AGA-piglets, but not in the SGA-piglets where blood glucose remained at birth level (Fig. 10A; green versus purple arrow). The explanation for this difference is twofold. The uptake of colostrum immediately after birth is very important in early development (Declerck et al. 2016; Devillers et al. 2011), and their larger size and higher vitality give the AGA-piglets more competitive access to the sow's teats to feed. Moreover, glycogen reserves (in the liver) at birth are considerably higher in AGA- than in SGA-piglets (Fig. 10B; in mass %), and these reserves are mobilized from birth onward in the AGA-piglets only (Fig. 10B; purple versus green arrows). Apparently, during the first hours of development, it becomes increasingly difficult for the SGA-piglets to keep up with their AGA-littermates because of energy- rather than neuromechanical deficits.

## General conclusions

The integration of all results presented in our former studies on the locomotion of term AGA- and SGA-piglets (Vanden Hole et al. 2017, 2018a, 2018b, 2019a, 2019b; Mielke et al. 2023) with the new data on body proportions, joint kinematics, and single-limb dynamics as given in the present synthesis, enables us to conclude the following:

Observed neuro-(loco)motor differences between AGA- and SGA-piglets during early development seem to boil down to an effect of size. Hence, size does matter!
Observed differences in vitality and mobility between AGA- and SGA-piglets during early development are probably not caused by differences in neuromechanical maturation but result from differences in the gestational- and early energy uptake and metabolism.
When SGA-piglets are used as a (loco)motor developmental model for human IUGR infants, the very short time window for neuromechanical maturation (< 4h), at least in locomotion, should be considered.
Still, some caution may be needed when interpreting the above results. Only SGA piglets that did walk from birth onward (yet, classified as “SGA/low vitality;” cf. “Methods” section and Vanden Hole et al. 2017 and 2018a for criteria) were used. Although inevitable in a study on the early development of locomotion, this could have biased the conclusions to some extent.

## Data Availability

The data underlying this article will be shared on reasonable request to the corresponding author.

## Notes

From the symposium “Biology at birth: the role of infancy in providing the foundation for lifetime success” presented at the annual meeting of the Society for Integrative and Comparative Biology annual meeting, January 3-7, 2023.
